# Asclepius with contemporary glasses introduces ‘Modern Radiology’—an open-access teaching resource

**DOI:** 10.1186/s13244-025-01953-3

**Published:** 2025-04-16

**Authors:** Minerva Becker

**Affiliations:** Diagnostic Department, Geneva University Hospitals, University of Geneva, Geneva, Switzerland

**Keywords:** eBook, Medical education, Radiology training, Didactic tools for radiology, Online education

## Abstract

**Abstract:**

The European Society of Radiology (ESR) initially developed the *ESR e-Book* as an open-access digital teaching resource aligned with the undergraduate European Training Curriculum for Radiology. Over time, this initiative evolved into *Modern Radiology*, a rebranded and expanded version that now serves not only medical students and educators but also residents and other healthcare professionals.

Unlike traditional textbooks, *Modern Radiology* follows a visually engaging and succinct format, emphasising key concepts through annotated images, attention points, hyperlinks, and self-assessment sections. It complements case-based and interactive learning methods, providing a systematic foundation for radiology education.

By continuously updating its content to reflect advancements in the field, *Modern Radiology* has been widely adopted by universities across Europe and beyond. Furthermore, several translations are in progress, ensuring its accessibility to a broader audience.

**Key Points:**

- *Modern Radiology* is an open-source, non-commercial teaching resource for undergraduate and postgraduate radiology teaching.
- It is a living document that follows the European Society of Radiology (ESR) Training Curriculum for Radiology.
- Its didactic and concise format facilitates the acquisition of basic knowledge and the preparation of interactive teaching and self-learning.
- It is widely used in universities across Europe and beyond, as well as by residents in training.

**Graphical Abstract:**

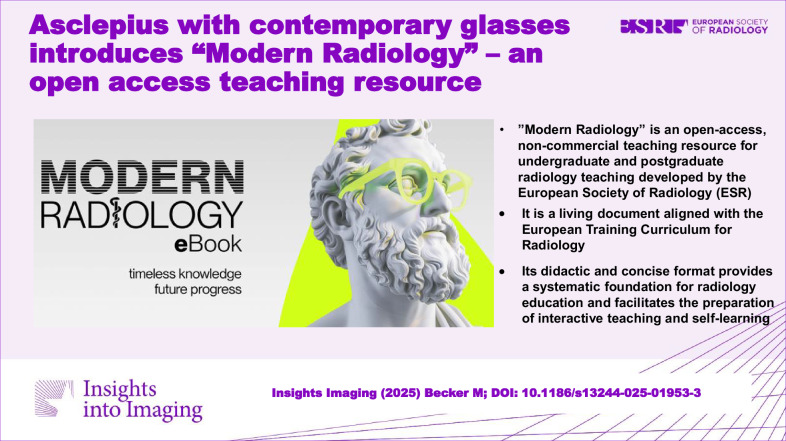

A few years ago, the European Society of Radiology (ESR) created and published the initial version of its *ESR e-Book* as an open-source digital teaching resource focusing mainly on the learning objectives of the undergraduate level of the *European Training Curriculum for Radiology* [1]. The current, rebranded version entitled *“Modern Radiology”* has been published with an enlarged scope, addressing not only medical students, teachers, and other medical professionals but also residents in training. The ESR provides *“Modern Radiology”* free of charge under the Creative Commons Non-Commercial Non-Derivatives 4.0 International License license. Its contents can be used for teaching purposes, provided that proper credit is given to the source.

Radiology may be taught in many ways according to the programmes of different universities and medical schools in each European country. At the pregraduate level, radiology teaching may either follow the form of a stand-alone discipline or as a ‘transverse’ discipline, being integrated into the undergraduate courses of other clinical specialties, e.g., surgery or internal medicine [2–4]. Education about medical imaging may therefore involve not only radiologists, but also academic teachers of different other medical specialties. One purpose of the *ESR e-Book* is to provide educators with a didactic resource created by European and non-European radiology experts, serving as a basis for their teaching courses.

Although the goal of the first version of the *ESR e-Book* was to provide a free online teaching tool for medical students and academic educators, it has constantly evolved in terms of topics and volumes until it encompassed over 28 chapters dealing with technically and clinically oriented topics. These are presented in over 2500 pages, based on the expertise of more than 100 recognised authors, and now also delve into topics related to postgraduate learning objectives.

Comments and feedback received from Europe and beyond have indicated that the depth and clarity of the didactic concept have been greatly appreciated and that the *ESR e-Book* fills a real gap not only for medical students but also for radiology residents and medical professionals from non-radiological specialties who wish to update or expand their knowledge in specific areas of medical imaging with which they are less familiar. These observations have been confirmed by over 160,000 downloads of the *ESR e-Book* during the past 2.5 years. Based on this rich feedback, the second edition of the *ESR e-Book* entitled *“Modern Radiology”* has been created and published online in order to cover topics not only related to the ‘undergraduate’ level of the *ESR Training Curriculum* but also the ‘postgraduate I and II’ levels, addressing and completing the educational needs in this rapidly evolving field [5].

Today, an abundance of innovative methods and web-based digital tools are available for radiology education. Many of these are designed to increase active engagement of students and residents with teachers and in team-based learning with peers, e.g., didactic courses with audience-response systems, the so-called flipped classroom approach, video tutorials, educational apps, embedded quick response (QR-) codes, or social media platforms [6, 7]. Case-based teaching strategies may include the analysis of complete digital image files based on anonymised DICOM image sets through which students and residents can scroll by themselves just as with a PACS workstation and then interactively discuss their observations with their teachers. Each of these methods may be useful in a given pedagogy, although specific skills or logistics are necessary for some of them [3, 4, 6, 7]. Nonetheless, recent feedback received from both members of the European radiological community and from undergraduate university programmes underlines the need for a concise but systematic, timely didactic overview of the entire field of medical imaging. The *ESR e-Book “Modern Radiology”* intends to serve this purpose, helping medical students and residents to acquire the basic knowledge which is necessary to benefit from interactive case-based teaching courses and to prepare for participation in advanced webinars or courses, e.g., in the context of the European School of Radiology (ESOR) or the ESR Connect platform. In addition, *Modern Radiology* aids residents in both developing a fundamental understanding of available imaging modalities and their clinical applications, as well as recognising key anatomical and pathological imaging features of common conditions across various organ systems.

The didactic concept of the *ESR e-Book* follows a simple format following the contemporary trend that favours image-based visual information over text to maintain attention because “an image is worth a thousand words”. The chapters are either based on organ- or technically related topics, with subtitles allowing to choose specific aspects of each topic. Concise information provides key points to memorise, as well as annotated images with captions, and hyperlinks allow the reader to connect with other chapters or complementary information. Each chapter also ends with a summary and contains a section for self-evaluation. However, *“Modern Radiology”* does not intend to replace the standard textbooks of radiology, which allow the reader to go into much more detail in each organ system; nor does it intend to replace radiological articles, which serve as a source of focussed, more in-depth learning. It can also neither serve as a radiologic encyclopaedia nor as a didactic case library.

The new graphical appearance of this second, rebranded and enlarged version includes the figure of Asclepius with fashionable glasses, symbolising the combination of classical medical teaching and contemporary style teaching. Due to its digital format, it is a ‘living’ teaching resource and only published online, thereby allowing the content to be regularly updated by the authors in this very dynamic medical discipline. Many prestigious universities and medical schools from Europe and beyond have already adopted the *e-Book* in its original English version for teaching purposes within their programmes. Translations into many different languages under the legal framework of ESR and respecting the rights of the authors of the original contents are currently in progress.

This initiative underscores ESR’s commitment to fostering high-quality, globally accessible radiology education, and on behalf of the Board of Directors of the ESR, I encourage the medical and radiological community of Europe and beyond to follow suit.
